# An up‐To‐Date Review of Elesclomol and Its Nano‐Formulations in Cancer Therapy

**DOI:** 10.1002/cnr2.70193

**Published:** 2025-04-07

**Authors:** Qi Wang, Feng Li, Amit K. Tiwari, R. Jayachandra Babu

**Affiliations:** ^1^ Department of Drug Discovery and Development Harrison College of Pharmacy Auburn Alabama USA; ^2^ National Institute on Drug Abuse, National Institutes of Health North Bethesda Maryland USA; ^3^ Department of Pharmaceutical Sciences College of Pharmacy, University of Arkansas of Medical Sciences Little Rock Arkansas USA

**Keywords:** anticancer, copper, cuproptosis, elesclomol, formulation, nanomedicine

## Abstract

**Background:**

Elesclomol (ES) is a promising anticancer compound that exerts its effects through multiple mechanisms. It acts as a copper (Cu(II)) ionophore, forming an ES–Cu complex within cancer cells and inducing a novel form of cell death called cuproptosis.

**Aim:**

To provide an up‐to‐date review on elesclomol and its nano‐formulations with a particular focus on cancer therapy.

**Sources:**

Literature was collected by manually searching in Pubmed, and Google Scholar, clinicaltrials.gov through March 2025.

**Content:**

This review provides an overview of the discovery and development of the ES molecule, including its physicochemical properties. New insights into the intracellular interactions of ES with copper and the mechanisms of copper transportation are then explained. The recent clinical outcomes of ES in cancer therapy, both as a monotherapy and in combination with paclitaxel or carboplatin, are summarized. While the initial clinical trials showed promise, more studies are focusing on the preclinical investigations of the ES–Cu complex. Nanomedicine‐based formulations have emerged as a strategy to enhance the intracellular delivery of ES as well as its therapeutic effects, with several ES–Cu nanomedicines currently under development. The recent nanoparticle delivery strategies of ES are discussed. This comprehensive review provides an up‐to‐date overview of the recent advancements in ES study, including its novel mechanism of action, clinical progress, and the potential of nanomedicine‐based approaches to improve its therapeutic efficacy in cancer treatment.

## 1 Introduction

### 1.1 Global Status of Cancer and Challenges in Current Therapeutics

Cancer remains one of the most critical global health challenges, with complicated therapeutic challenges [1]. Socioeconomic aspects determine cancer's disproportionate impact on populations.

High‐income countries experience the greatest absolute increase in incidence, but low‐ and middle‐income countries face the steepest proportional increases in both incidence and mortality due to limited access to early detection and treatment services [1]. Though cancer treatment has advanced significantly, numerous issues still exist.

#### 1.1.1 Drug Resistance and Tumor Heterogeneity

Multiple cancers with genetic flexibility and cellular heterogeneity complicate therapies. Although targeted monotherapies show promise, they typically fail as single agents due to drug resistance or a failure to treat numerous mutations in a tumor site. Patients with chronic myeloid leukemia, for example, who were treated with targeted medicines such as imatinib, eventually developed resistance.

#### 1.1.2 Toxicity and Safety Concern

Combination therapies have been utilized but can lead to increased toxicity and adverse effects [2].

Even advanced therapies like immunotherapy and precision oncology can cause severe side effects such as immune‐related adverse effects or hyperprogressive disease (accelerated tumor growth). In many situations, chemotherapy‐induced side effects such as myelosuppression or neuropathy are still poorly managed. Toxicology processes, which frequently overlap with the efficacy mechanisms of drugs, make it difficult to limit effects while maintaining efficacy [3].

#### 1.1.3 Disparities in Access

Socioeconomic inequalities restrict access to diagnostic instruments, conventional remedies, and cutting‐edge therapies in low‐ and middle‐income nations. Participation in clinical trials is disproportionately biased towards high‐income populations, resulting in the underrepresentation of other demographic groups. This disparity leaves these populations without access to personalized therapeutic options or sufficient resources for representation in research [4].

To address these challenges, many improvements need to be followed, such as improving early detection through affordable screening programs, overcoming drug resistance, and improving the safety profile of treatments. Addressing disparities through equitable healthcare policies and inclusive clinical trials is critical for global progress against cancer.

### 1.2 Physicochemical Properties of ES

**FIGURE 1:**
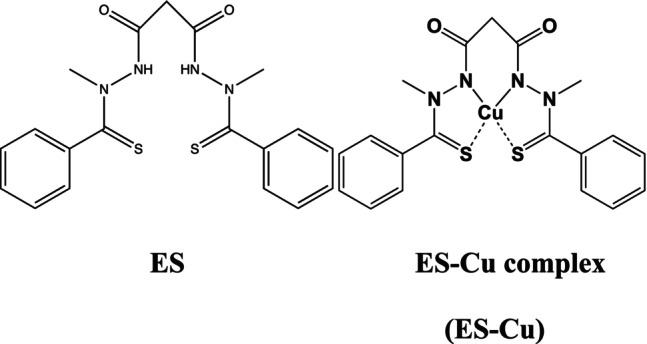
The structures of ES and ES–Cu complex.

ES (N‐malonyl‐bis(N′‐methyl‐N′‐thiobenzoylhydrazide); STA‐4783; ES) is an investigational anticancer drug that was first developed by Synta Pharmaceuticals. ES molecular weight is 400.52 g/mol and is practically insoluble in water. ES–Cu complex molecular weight is 462.1 g/mol. It can dissolve in DMSO (80 mg/mL at 25°C). ES is a copper ionophore and can bind Cu(II) to form the ES–Cu complex. The structures of ES and the ES–Cu complex, and the physicochemical properties of ES are shown in Figure 1 and Table 1, respectively.

**TABLE 1:** The physicochemical properties of ES. The information was predicted by http://www.swissadme.ch/index.php, https://chemaxon.com/calculators‐and‐predictors, and https://go.drugbank.com/drugs/DB05719.

Physicochemical property	
Formula	C19H20N4O2S2
Molecular weight	400.52 g/mol
Num. heavy atoms	27
Num. arom. heavy atoms	12
Fraction Csp3	0.16
Num. rotatable bonds	10
Molar Refractivity	112.11
Topological polar surface area	128.86 Å^2^

Lipophilicity	
Log *P*_o/w_ (iLOGP)	2.16
Log *P*_o/w_ (XLOGP3)	2.89
Log *P*_o/w_ (WLOGP)	2.05
Log *P*_o/w_ (MLOGP)	2.92
Log *P*_o/w_ (SILICOS‐IT)	3.22
Consensus Log *P*_o/w_	2.65

Druglikeness	
Lipinski	Yes
Ghose	Yes
Veber	Yes
Egan	Yes
Muegge	Yes

### 1.3 Pharmacological Activity of ES

ES promotes apoptosis in cancer cells by producing reactive oxygen species (ROS) and inducing oxidative stress. ES is a copper‐transporting drug that can chelate copper ions to enhance oxidative stress in cancer cells, resulting in apoptosis [5, 6]. Previous in vitro studies showed that ES induced a rapid increase in the production of ROS, as well as the stimulation of a transcriptional gene profile with oxidative stress response. Inhibition of oxidative stress by an antioxidant N‐acetylcysteine limited the stimulation of apoptosis, demonstrating that ROS production was responsible for the proapoptotic activity induced by ES [7].

A recent study has first demonstrated that ES induces cuproptosis, which is a new copper‐dependent non‐apoptotic cell death pathway [8]. The cuproptosis induced by ES is highly dependent on intracellular mitochondrial metabolism and copper ions (Cu(II)) transport [9, 10, 11]. ES can chelate with Cu(II) and then transport Cu(II) inside cell membranes. The Cu(II) then bonds to fatty acylated components in the tricarboxylic acid (TCA) cycle, which further results in protein aggregation and iron–sulfur cluster protein reduction, followed by cell death [12]. A mitochondrial enzyme called ferredoxin 1 (FDX1) has been demonstrated to be the direct target of ES to promote cuproptosis and is associated with reducing Cu(II) to Cu(I) inside cells [13, 14]. The mechanism of how the ES–Cu complex interacts with FDX1 was first illustrated in a recent research study in 2019 [14]. In this study, researchers have shown that ES induces cytotoxicity through FDX1 in two different pathways. First, ES can act as an upstream regulator of mitochondrial function. It directly binds to the reduced FDX1 and prevents it from exerting its activity in Fe‐S cluster production. Second, ES can bind Cu(II) to form an ES–Cu complex. The ES–Cu complex acts as a neo‐substrate for reduced FDX1, which then results in oxidative stress and the production of Cu(I). This copper‐dependent cell death cannot be inhibited by apoptosis or ferroptosis inhibitors and has been demonstrated to be cuproptosis in another research paper published in 2022 by the same research group [8, 14].

### 1.4 Development and Synthesis of ES

Synta Pharmaceuticals has revealed the development of ES in a research paper published in 2013 [15]. In this paper, a series of *N*′^1^,*N*′^3^‐dialkyl‐*N*′^1^,*N*′^3^‐di(alkylcarbonothioyl) malonohydrazides were synthesized and screened. Researchers screened these compounds in Synta's compound library, and structure–activity relationship studies, Hsp70 induction assays, and a cytotoxicity assay using a multidrug‐resistant cancer cell line (MES‐SA/Dx5) were performed. These efforts then led to the discovery of ES [15].

**FIGURE 2:**
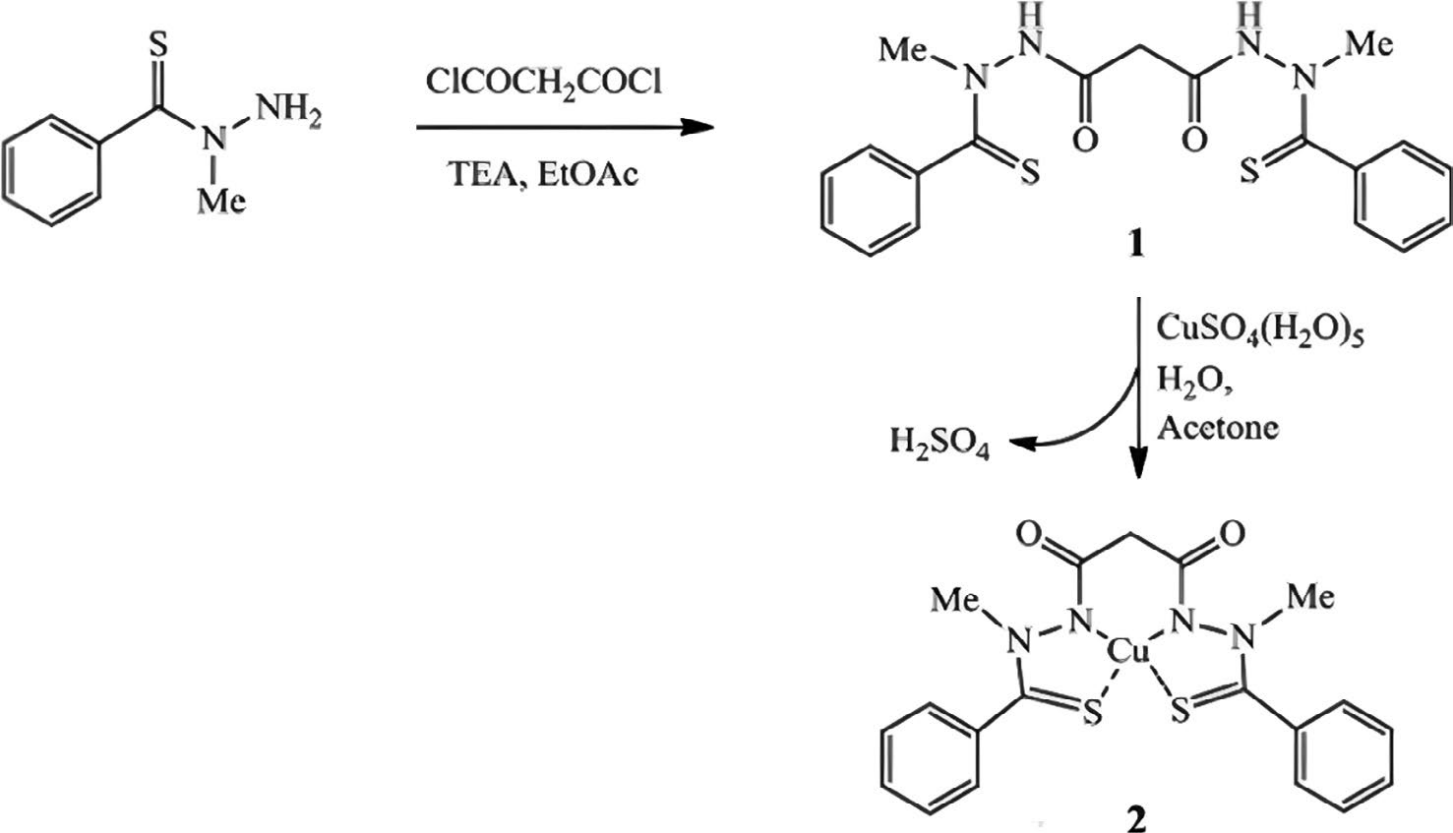
The synthesis of ES and ES–Cu complex. Reprinted with permission of Ref. [17]. Copyright 2013 Elsevier Inc.

Different synthesis methods of ES were revealed in several studies [6, 15, 16, 17]. Synta Pharmaceuticals reported a synthesis method of ES in 2013 [17]. Two equivalents of N‐methylbenzothiohydrazide reacted with one equivalent of malonyl chloride in ethyl acetate through direct acylation to yield the final product ES. This method was easier to conduct in reaction and purification compared to a report published also by Synta Pharmaceuticals in 2013 [15]. It avoided using explosive perchloric acid or N,N′‐dicyclohexylcarbodiimideor 1‐ethyl‐3‐(3‐dimethylaminopropyl) carbodiimide. ES–Cu complex was synthesized by mixing ES and copper sulphate pentahydrate in acetone. The synthesis of ES and ES–Cu complex is illustrated in Figure 2.

**FIGURE 3:**
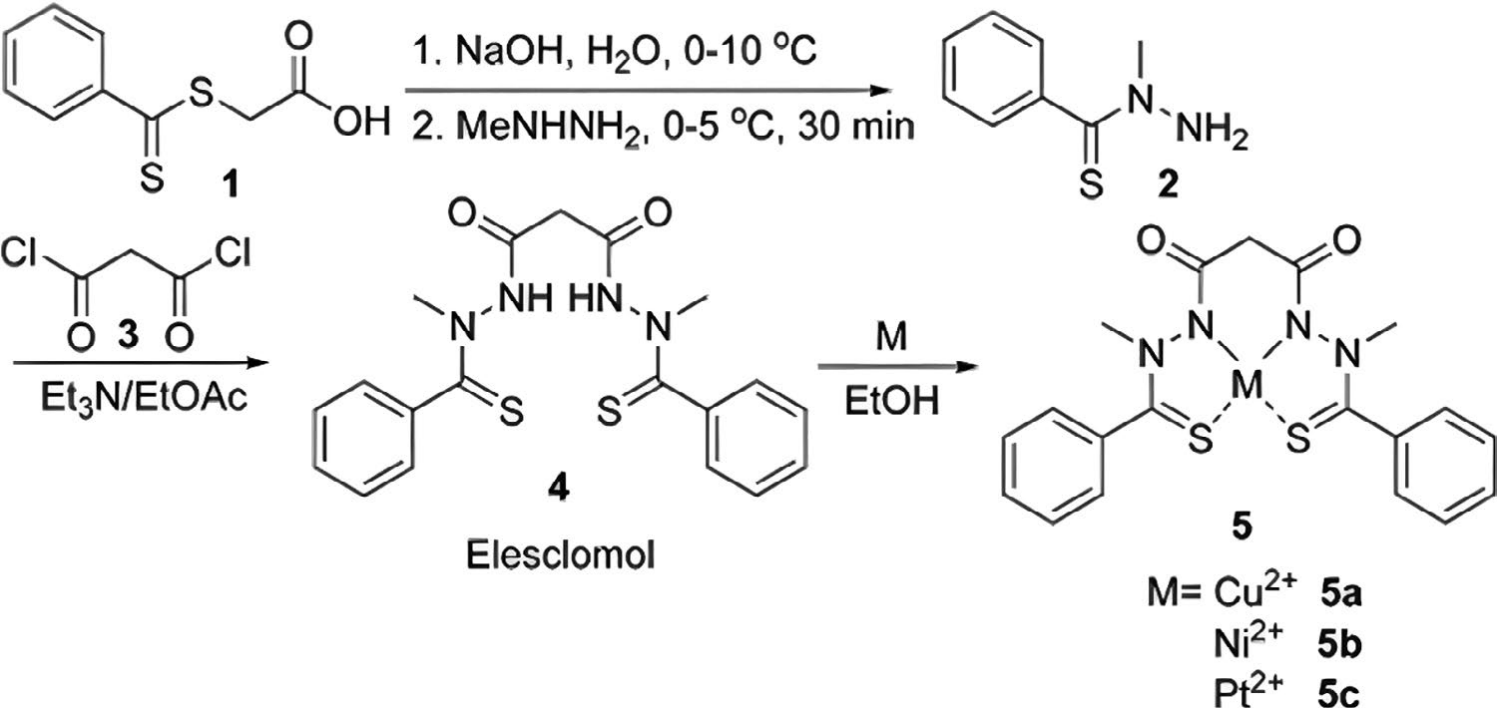
Structure of ES (**4**) and the reaction scheme for its synthesis and intermediates for the preparation of its complexes with Cu^2+^, Ni^2+^ and Pt^2+^ (**5a**, **5b**, and **5c**, respectively). Reprinted with permission of Ref. [6]. Copyright 2013 Elsevier Inc.

In another report published in 2013, Yadav et al. first synthesized N‐methylbenzothiohydrazide by mixing S‐(thiobenzoyl)thioglycolic acid and methylhydrazine trifluoroacetic acid salt, and the reaction was maintained between 0°C and 10°C. N‐methylbenzothiohydrazide was obtained and then purified by 0.1 M aqueous NaOH and heptane. Next, N‐methylbenzothiohydrazide was mixed with malonyl chloride, and the reaction was maintained between 0°C and 10°C, which was later quenched by H_2_O. The reaction product, ES, was then purified by column chromatography on silica gel and characterized by ^1^H NMR and ^13^C NMR. This study also introduced a synthesis method of the ES–Cu complex by mixing ES and CuCl_2_ in ethanol, and the reaction product, the ES–Cu complex, was collected by filtration using methylene chloride [6]. The structure of ES and the reaction scheme for ES synthesis and intermediates for the preparation of ES complexes with Cu^2+^, Ni^2+^, and Pt^2+^ is shown in Figure 3.

### 1.5 The Preclinical and Early Clinical Results of ES

ES has shown promising results in several preclinical studies. ES can enhance sensitivity to copper‐mediated cell death in proteasome inhibitor resistant cancer cells [14]. In another research study, Modica‐Napolitano et al. first demonstrated that the ES–Cu complex has a direct effect on bioenergetic function in isolated mammalian mitochondria and may promote uncoupling and/or blocking mitochondrial oxidative phosphorylation [7]. Garza et al. explained the mechanism of ES distributing copper in cells and demonstrated that the ES–Cu complex can transport Cu(II) to Ccc2 and bypass both Atx1 and Grx1, indicating that this new mechanism has the potential to benefit copper deficiency disorders [18]. Liu et al. demonstrated that [^64^Cu][Cu(ES)] enhanced efficacy in inhibiting hypoxic solid tumors compared to both [^64^Cu][Cu(ATSM)] and [^64^Cu]CuCl_2_ in in vitro and in vivo studies [19].

Several clinical trials were conducted either using ES alone or in combination with paclitaxel or carboplatin for cancer treatment. ES has a high safety profile in clinical results; however, ES has not reached endpoints in several clinical trials [20, 21, 22, 23, 24, 25, 26, 27, 28]. The possible reasons will be discussed later. There have been no clinical trials of ES–Cu complex so far. The new mechanisms of ES in cancer cells with different metabolism activities, which were revealed recently, may provide some guidance in future clinical trials of ES or ES–Cu complex [8, 13, 14].

## 2 Mechanisms of ES in Inducing Anticancer Activities

### 2.1 Structure of ES and ES–Cu Complex

ES is a bis(thiohydrazide) amide and has ketone, hydrazide, and thiosemicarbazone groups in its structure which allow it to chelate Cu(II) with a molar ratio of 1:1. The binding of Cu(II) with the loss of two protons from two nitrogen atoms of ES produces a neutral ES–Cu complex. ES can also form complexes with other redox‐inactive metal ions, such as Ni(II) and Pt(II). However, these redox‐inactive ES–Ni and ES–Pt complexes showed almost inactive inhibition in K562 cell growth compared to the ES–Cu complex, indicating a biologically reducible redox‐active metal ion was necessary for the ES–Cu complex to induce cytotoxicity. Reoxidation of Cu(I) after the ES–Cu complex entering cells could then induce oxidative stress by generating ROS. In a liquid chromatography‐mass spectrometry study, ES was shown to bind Cu(II) much more strongly than Ni(II). ES binds Cu(II) with extremely high affinity (*K*_a_ = 1017.1) at pH 7.4 [6]. The mechanism of how Cu(I) is released from the ES–Cu complex in cells will be explained in the next section.

### 2.2 New Mechanism of Intracellular Copper Release From ES–Cu Complex

Mitochondrial metabolism is crucial for adaptability to proteotoxic stress. Cancer cells, mouse xenografts, and patient‐derived tumor samples that switch mitochondrial metabolism from glycolysis to oxidative phosphorylation (OXPHOS) are resistant to proteasome inhibitors (PI) such as bortezomib. These PI‐resistant samples with shifted high OXPHOS metabolism rates are sensitive to ES, which can induce a copper‐dependent cell death. These discoveries led to the identification of the ES direct target FDX1, which is a mitochondrial reductase. ES–Cu complex serves as a neo‐substrate of reduced FDX1, which can promote the production of free Cu(I) and oxidative stress. It demonstrated that FDX1 mediates the reduction of Cu(II) and the production of Cu(I). These results also may provide new strategies for PI‐resistant treatment [14]. This study explained that free Cu(I) can be released from the ES–Cu complex through an FDX1‐mediated copper‐dependent cell death pathway.

**FIGURE 4:**
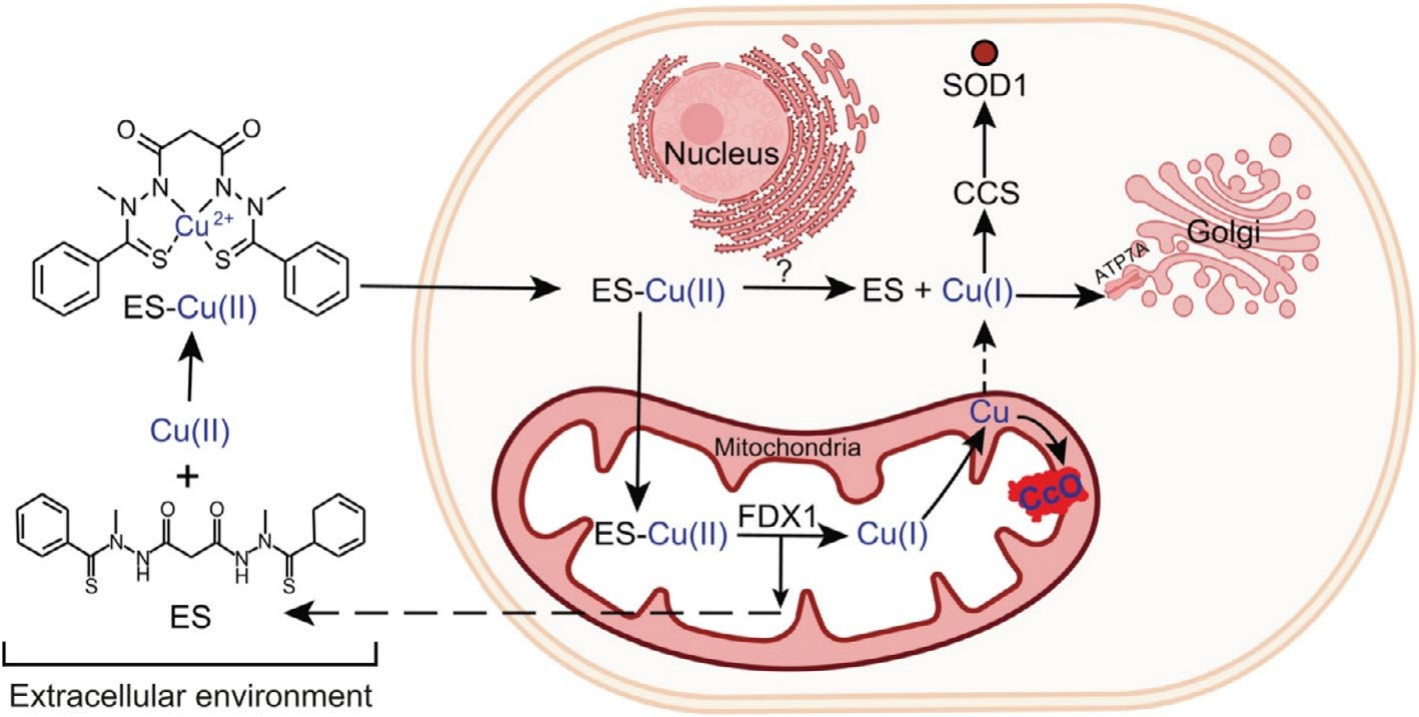
The mechanism of FDX1‐dependent and FDX1‐independent Cu(I) release from ES–Cu complex. Reprinted with the permission of Ref. [13]. Copyright 2023 the Author(s). Published by PNAS.

However, a recent study has revealed that the mechanism of Cu(I) release from the ES–Cu complex in different subcellular compartments is both FDX1‐dependent and FDX1‐independent by using a combination of genetic, biochemical, and cell‐biological approaches. These manners of ES transporting Cu(II) are different from other copper transporters tested in the study. Zulkifli et al. proved that electron transfer from reduced FDX1 to the ES–Cu complex can lead to the formation of oxidized FDX1 and ES–Cu(I) species. Then, they evaluated ATP7A translocation with a lower dosage of the ES–Cu complex. The data showed that FDX1 is not required for ATP7A trafficking in response to increased levels of Cu(I) in the cytoplasm. They observed released Cu(I) in FDX1 absent Ctr1^−/−^ and Ctr1^−/−^Fdx1^−/−^ cells. ES–Cu complex treatment reduced the abundance of the CCS protein but stimulated SOD1 activity in both Ctr1^−/−^ and Ctr1^−/−^Fdx1^−/−^ cells. These results demonstrated that Cu(I) release outside of mitochondria in the cytoplasm is FDX1‐independent. However, this research study did not elucidate how Cu(I) is released from the ES–Cu complex outside mitochondria [13]. Figure 4 illustrates FDX1‐dependent and FDX1‐independent Cu(I) release. There is a need for more research to gain a deeper understanding of intracellular copper transport. This study provides new information for copper disorder research.

### 2.3 ES Inducing Cell Death Through Cuproptosis

**FIGURE 5:**
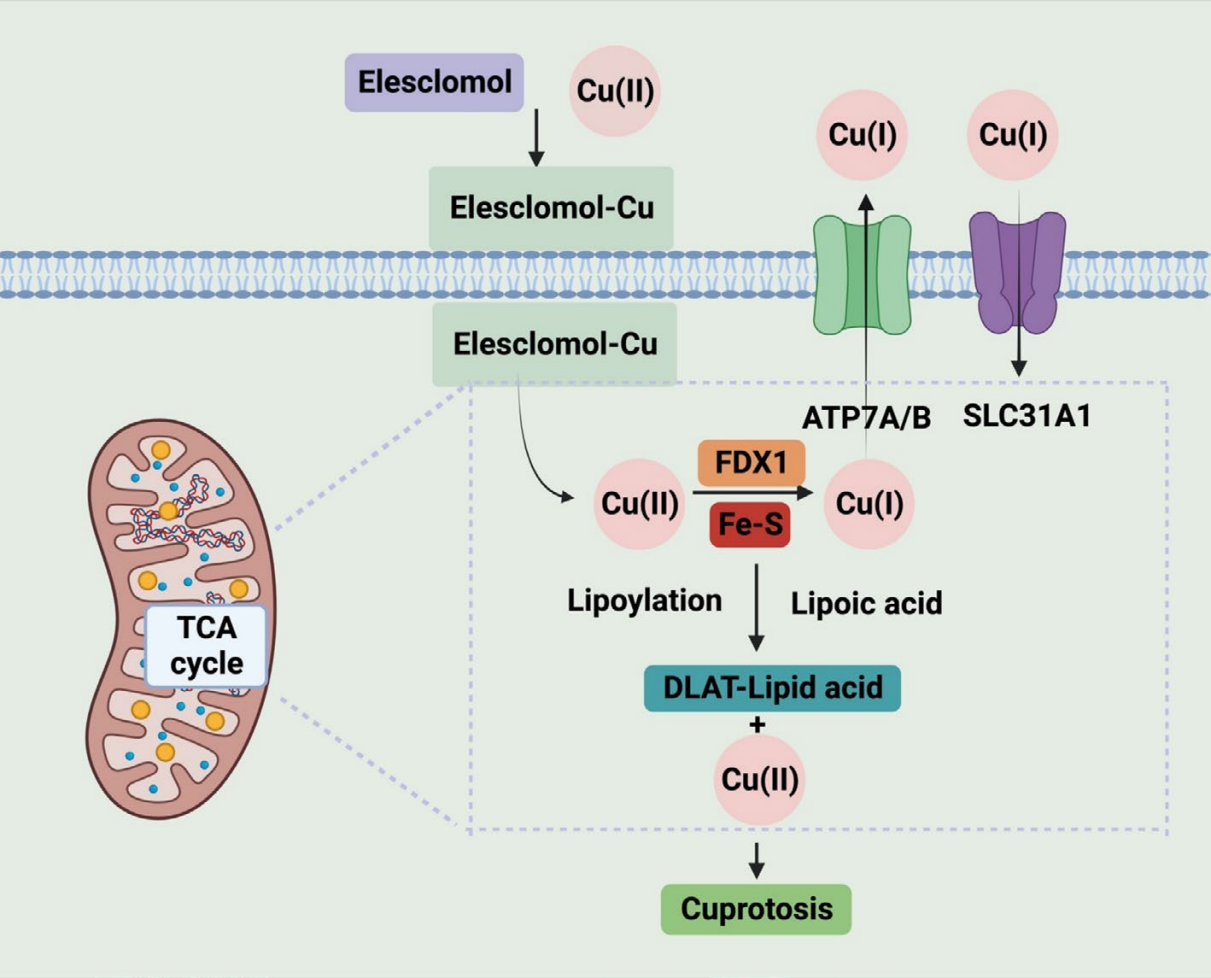
The illustration of ES‐induced cuproptosis in cancer cells (Created with Biorender.com).

A recent study has demonstrated ES can induce cell death through cuproptosis. Tsvetkov et al. proposed in 2022 that cuproptosis is a new copper‐dependent cell death manner and is regulated by mitochondrial FDX1‐mediated protein lipoylation [8]. Cuproptosis is different from other cell death pathways such as apoptosis or ferroptosis and is dependent on intracellular copper accumulation. ES can shuttle extracellular copper inside cells to dysregulate copper balance and further induce cell death. Intracellular Cu(II) concentration increases due to the transport of ES, followed by FDX1‐mediated mitochondrial proteotoxic stress [14]. Cuproptosis‐induced cell death is not mainly dependent on the ROS generated after ES–Cu treatment. Researchers treated cells with a few antioxidants, such as N‐acetylcysteine, α‐tocopherol, ebselen, and JP4‐039, and all of these antioxidants did not effectively scavenge the cell death induced by ES–Cu. However, glutathione (GSH) can reduce the toxicity of ES–Cu due to its ability to chelate intracellular copper rather than its antioxidative ability. This result indicates that the cell death induced by ES–Cu is copper‐dependent. ES binds to Cu(II) to form an ES–Cu complex, and this ES–Cu complex directly targets FDX1, which can reduce Cu(II) to Cu(I) and then release the free Cu(I) inside mitochondria with an increase of oxidative stress, leading to an inhibition of Fe–S cluster synthesis and reduction of Fe–S cluster proteins. Tsvetkov et al. performed genome‐wide CRISPR/Cas9 knockout screen combined with metabolic and biochemical assays to prove that copper can enhance mitochondrial protein lipoylation. Copper‐induced cell death is mediated by protein lipoylation. The levels of lipoylated TCA enzymes are increased in TCA‐active cells, where copper can directly bind to the lipoyl moiety. This process leads to lipoylation‐dependent oligomerization of dihydrolipoamide S‐acetyltransferase (DLAT). The gel electrophoresis results in their group showed that the levels of DLAT oligomers increased in ES‐sensitive cells after ES treatment. However, the increased levels of DLAT oligomers only occurred in ES‐insensitive or FDX1 knockout cells after treatment with a higher concentration of ES. It indicated that the toxicity induced by ES was associated with the loss of Fe‐S cluster proteins and was FDX1‐dependent. The process of FDX1 reducing Cu(II) to Cu(I) is involved in the TCA cycle. In summary, Peter Tsvetkov et al. were the first group to prove that the ES–Cu complex induced cell death, which was due to copper toxicity. When depleting intracellular copper, ES alone did not induce cell death. This result suggested that the ES–Cu complex induced a copper‐dependent cell death, and excess copper causes lipoylated proteins to aggregate, which is associated with mitochondrial metabolism [13, 14, 29]. An illustration of the cuproptosis mechanism is shown in Figure 5. A comparison of the cuproptosis mechanism with other anticancer therapies can be found in Table 2.

**TABLE 2:** Comparison of cuproptosis mechanism with other anticancer therapies.

Mechanism	Cuproptosis inducer: Elesclomol	Apoptosis inducers	Ferroptosis inducers	ROS inducers	Targeted therapies (e.g., TKIs)
Primary mechanism	Induces cuproptosis, a copper‐dependent cell death pathway through mitochondrial dysfunction and protein aggregation in the TCA cycle [27, 30].	Induces programmed cell death via caspase activation and DNA damage (e.g., paclitaxel, cisplatin) [28].	Causes iron‐dependent lipid peroxidation leading to cell death (e.g., erastin, RSL3) [31].	Increases reactive oxygen species (ROS) to induce oxidative stress and apoptosis (e.g., doxorubicin) [27, 28, 31].	Blocks specific signaling pathways that drive cancer growth (e.g., imatinib, erlotinib) [28].
Target	Copper ion transport into mitochondria, leading to mitochondrial dysfunction and proteotoxic stress [27, 30].	DNA, microtubules, or pro‐apoptotic proteins [28].	Iron metabolism and lipid peroxidation [31].	ROS generation through redox cycling of copper ions (Cu(II) to Cu(I)) [27, 28, 31].	Specific tyrosine kinases or signaling molecules [28].
Cell death pathway	Cuproptosis—non‐apoptotic, copper‐dependent mitochondrial protein aggregation [27, 30].	Apoptosis—caspase‐mediated, DNA fragmentation, and cell death [28].	Ferroptosis—iron‐dependent accumulation of lipid peroxides [31].	ROS‐induced apoptosis via oxidative stress [27, 28, 31].	Inhibition of growth signals leading to apoptosis or cell cycle arrest [28].
Metabolic focus	Targets mitochondrial metabolism, particularly in cells with high oxidative phosphorylation (OXPHOS) [27, 30, 31].	Not specific to metabolic pathways; targets proliferating cells [28].	Disrupts iron metabolism and lipid homeostasis [31].	Alters redox balance via ROS production [27, 28, 31].	Alters signaling pathways without direct impact on metabolism [28].
Copper dependency	Requires copper ions to induce cytotoxicity; forms ES–Cu complex that disrupts mitochondrial function [27, 30].	Copper‐independent; relies on DNA or protein damage for apoptosis induction [28].	Iron‐dependent; no role for copper in ferroptosis induction [31].	Copper is involved in redox cycling but not essential for all ROS‐inducing therapies [27, 28, 31].	Copper‐independent; targets specific proteins or receptors in cancer cells [28].
Resistance mechanism	Effective against proteasome inhibitor‐resistant cancers with high OXPHOS activity [27, 31].	Resistance can develop through mutations in apoptotic pathways or drug efflux pumps [28].	Resistance can occur through increased antioxidant defenses or altered iron metabolism [31].	Resistance can develop due to increased antioxidant defenses like glutathione production [27, 28, 31].	Resistance can arise from mutations in target proteins or compensatory signaling pathways [28].

## 3 Recent Preclinical Applications of ES in Cancer Therapy

### 3.1 ES In Monotherapy

#### 3.1.1 ES In GNAQ/11‐Mutant Uveal Melanoma

A recent research study by Li et al. showed that ES can be used as an antagonist against guanine nucleotide‐binding protein G(q) subunit alpha or guanine nucleotide‐binding protein G (11) subunit alpha (GNAQ/11) mutation in uveal melanoma (UM) [32]. UM is distinguished from cutaneous melanoma due to its mutations in GNAQ and GNA11. GNAQ and GNA11 proteins are α subunits of a heterotrimeric G protein, which plays an important role in G protein‐coupled receptor (GPCR) signaling [32]. UM is still lethal due to the lack of any effective targeted therapy or immunotherapy [33, 34]. Treating GNAQ/11‐mutant cells with ES generated a significant increase of ROS and stimulation of nuclear translocation of NRF2, while treating GNAQ/11‐wild‐type cells with ES could not induce a similar result. GSEA of RNA‐seq was performed to show that downregulation of oxidative phosphorylation and peroxisome and upregulation of hypoxia and apoptosis signaling happened in ES‐treated GNAQ‐mutant 92.1 cells. The data indicated GNAQ‐mutant cells could have a different metabolic pathway than those wild‐type cells. Next, the researchers found that ES could promote phosphorylation of YAP and LATS1 without impacting the ERK and AKT pathways in GNAQ/11‐mutant UM cells. This data suggested that inhibiting the MEK and YAP signaling pathways together could make GNAQ/11‐mutant cells more sensitive to MEK inhibitors. By mediating copper entry into mitochondria and inducing ROS, ES selectively kills GNAQ/11‐mutant UM cells by activating the LATS1‐YAP axis. They also proved that treating GNAQ/11‐mutant UM cells with binimetinib and ES together could increase cytotoxicity and sensitivity. Since UM responds poorly to MEK inhibitors, this study reported that ES could provide a potential administration strategy to synergize with MEK inhibitors such as binimetinib by reducing resistance to MEK inhibitors in GNAQ/11‐mutant UM [32].

#### 3.1.2 ES In Proteasome‐Resistant Cancer Cells

Studies showed that the PI‐resistant state is associated with the downregulation of the 19S regulatory complex of the proteasome [14, 35, 36]. In a research study published by Peter Tsvetkov et al., they named this state as the Lo19S state. They proved that there were statistically significant changes in tricarboxylic acid cycle (cis‐aconitate) and redox (NAD, NADH, GSSG) metabolites and some metabolites related to the TCA cycle in Lo19S state cells treated with PIs. The results indicated that proteasome inhibitor treatment causes alterations in mitochondrial gene expression and metabolite levels in Lo19S state cells and in vivo models, indicating a shift in mitochondrial metabolism occurred. This showed that the treatment of PIs caused the Lo19S state cells to use OXPHOS instead of glycolysis, which was used in control cells. The researchers named this metabolism‐shifted state as the Hi‐Mito state. To further confirm it, the researchers also cultured the Lo19S state cells in galactose media to let the cells use OXPHOS rather than glycolysis. The results showed that the cells cultured in galactose were resistant to PIs. However, these PI‐resistant cells were extremely sensitive to ES, indicating an alternative strategy for PI‐resistant cancer therapy. This study was the first to prove that PI‐resistant cancer cells were associated with a shift in metabolism from glycolysis to OXPHOS, and ES was able to induce copper‐dependent cell death in PI‐resistant cancer cells. It provided an alternative administration strategy for PI‐resistant cancer therapy [14].

### 3.2 ES In Combination With Ferroptosis Inducers in Primary Liver Cancer

**FIGURE 6:**
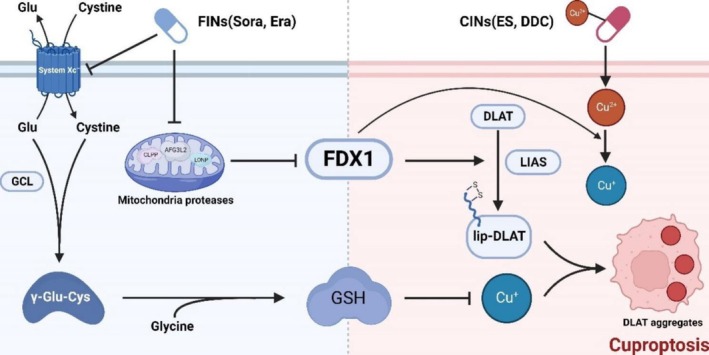
The illustration of ferroptosis inducers enhanced cuproptosis induced by copper ionophores in primary liver cancer. Reprinted from Ref. [37]. Copyright 2023, The Authors. BMC Part of Springer Nature.

A recent study by Wang et al. reported that ferroptosis inducers could promote cuproptosis through protein lipoylation activation and GSH synthesis suppression (Figure 6). They proposed that the combination of ES–Cu complex and ferroptosis inducers such as sorafenib could be a potential therapeutic method for liver cancer treatment. In this study, the researchers first proved that two ferroptosis inducers, sorafenib and erastin, could upregulate intracellular lipid peroxidation by suppressing mitochondrial matrix‐related proteases‐mediated FDX1 protein degradation and decreasing intracellular GSH concentration via inhibiting cystine importing in HCC and ICC cells. This step could promote the protein lipoylation process and the transfer of Cu(I). The GSH with a decreased concentration induced by ferroptosis inducers could further increase Cu(II) concentration, followed by an enhanced aggregation of lipoylated proteins [37]. Tsvetkov et al. reported before that ES induced cuproptosis, which was mediated by protein lipoylation [8]. The enhanced protein lipoylation induced by ferroptosis inducers could then enhance cuproptosis induced by ES. The researchers validated their results in liver cancer in vivo.

An in vivo study demonstrated that the combination of ES and ferroptosis inducer sorafenib had an anti‐tumor effect in liver cancer in nude mice xenografted by subcutaneous inoculation with MHCC‐97 H and QBC939 cells. This study provided a promising treatment solution for liver cancer treatment [37]. A summary of various preclinical studies on ES in cancer therapy is shown in Table 3.

**TABLE 3:** A brief summary of preclinical applications of ES.

Cancer type	Efficacy	Mechanism	Application
Melanoma [38]	Broad spectrum anti‐tumor activity	ROS generation and mitochondrial apoptosis	Single‐agent treatment and combination with paclitaxel
Ovarian cancer [20, 27]	Enhanced sensitivity to paclitaxel	Oxidative stress increase	Combination with paclitaxel
Leukemia [27, 31]	Effective against drug‐resistant cells	Copper‐dependent cell death	Single‐agent treatment or combination therapy
Cancer stem cells [27]	Reduced sphere formation	Mitochondrial metabolism targeting	Targeting cancer stem‐like cells resistant to standard therapies
Lung cancer [27]	The sensitivity of cisplatin‐resistant lung cancer cells to elesclomol is closely related to mitochondrial metabolism	ROS induction and intrinsic apoptotic cascade activation	Single‐agent
Colorectal cancer [27, 31]	Effective in targeting cancer cells through mitochondrial dysfunction	Induction of cuproptosis	Copper‐dependent ferroptosis induction
Hypoxia‐resistant tumors [20, 27, 31]	Limited efficacy under hypoxic conditions where glycolysis predominates	Targeting tumors reliant on oxidative phosphorylation under normoxic conditions	Targeting tumors reliant on oxidative phosphorylation

## 4 Clinical Trials of ES Alone and in Combination With Other Drugs in Cancer Therapy

### 4.1 Clinical Outcomes

A summary of the clinical trials of ES and ES in combination with other drugs is presented in Table 4. In a phase 3 clinical study, patients with advanced melanoma were treated with ES in combination with paclitaxel. The 651 participants in the research were given either ES + 80 mg/m^2^ paclitaxel (ELPAC) or paclitaxel alone. The study did not reach its primary endpoint of progression‐free survival (PFS) with > 90% with a 2‐month improvement, and ELPAC demonstrated an increase in mortality occurrences as well as common adverse events. The experiment was terminated due to an imbalance in overall deaths in patients with high lactate dehydrogenase (LDH) levels [39, 40].

**TABLE 4:** The clinical trials of ES (Information was obtained from https://clinicaltrials.gov/ (accessed on March 1, 2025).

Clinical trial/drug	Condition	Status	Phase	Participants	Clinical trials ID
ES/Paclitaxel	Melanoma	Terminated	Phase 3	630	NCT00522834
ES Sodium	Acute myeloid leukemia	Unknown	Phase 1	36	NCT01280786
ES Sodium	Metastatic solid tumors	Suspended	Phase 1	30	NCT00827203
ES Sodium/Paclitaxel	Recurrent or persistent platinum‐resistant ovarian, fallopian tube or primary peritoneal cancer	Completed	Phase 2	58	NCT00888615
ES Sodium/Docetaxel	Prostate cancer	Completed	Phase 1	34	NCT00808418
ES	Soft tissue sarcoma	Completed	Phase 2	80	NCT00087997
ES/Paclitaxel	Neoplasms	Completed	Phase 1	50	NCT00088114
ES/Paclitaxel	Melanoma	Completed	Phase 1/2	103	NCT00084214
ES/Paclitaxel/Carboplatin	Stage IIIB or stage IV non‐small cell lung cancer (NSCLC)	Completed	Phase 1/2	86	NCT00088088

In a phase 2 trial of IV ES with weekly paclitaxel treatment in platinum‐resistant recurrent ovarian cancer patients, acceptable tolerability was achieved but no satisfactory response was observed. With no reports of grade 4 toxins, the combination therapy had a median of three cycles and an overall median survival of 13.3 months. The study indicated that the combination was tolerable but not worth further investigation [20].

In a phase 2 study, the goal was to assess the safety and tolerability of ES sodium in patients with advanced myeloid leukemia (AML) by providing a weekly 60‐min infusion. The first dosage was 200 mg/m^2^. The efficacy of ES sodium was evaluated using bone marrow aspirate. Six patients were given one complete cycle of ES sodium, with a median length of 22 days. However, one patient died from pneumonia after only 1 day, and the project was terminated after the second dose level (400 mg/m^2^) due to patient recruiting difficulties [21].

A phase 1 clinical trial was conducted to determine the maximum tolerated dose, toxicity profile, and pharmacokinetics of ES when combined with paclitaxel in adults with resistant solid tumors. The drugs were administered as a 3‐h intravenous infusion, with initial dosages of 44 mg/m^2^ for ES and 135 mg/m^2^ for paclitaxel. There were 35 participants in the study who received eight different dose levels of ES/paclitaxel. In patients, severe toxicity increased with ES dose increasing, with 438 mg/m^2^ being the maximum tolerable dose. Partial responses were found in patients with Kaposi's sarcoma and ovarian cancer, both with disease progression while receiving prior paclitaxel treatment. The combination showed tolerability and a dose‐dependent response to ES in patients [41].

Another clinical trial was conducted to evaluate the efficacy of ES 213 mg/m^2^ in combination with paclitaxel 80 mg/m^2^ in patients with metastatic melanoma. The combination resulted in a twofold increase in median PFS and a 41.7% reduction in disease progression or death. The E + *P* group had a 15% response rate, while the paclitaxel group had a 3% response rate. The median survival time was 11.9 months, with 68% of patients switching to combination therapy after disease progression. The treatment exhibited an acceptable level of toxicity and showed promising overall survival (OS) outcomes [24].

### 4.2 Safety and Efficacy Considerations With ES Based on Current Clinical Studies

Several factors can explain ES's failure to meet clinical trial outcomes, including effectiveness issues, safety concerns, patient selection, and pharmacokinetic limitations. These factors have impeded the clinical performance and outcomes of excellent preclinical research [20, 21, 24, 39, 40, 41].

Finally, compared to treating paclitaxel alone, several ES clinical trials did not show a significant increase in PFS with the combination of ES and paclitaxel. It suggested that the results did not meet their primary outcomes. There are several reasons why ES fails in clinical trials, and these findings should considerably guide future research.

#### 4.2.1 Patient Selection and Metabolic Variability

One reason for the failure of ES clinical trials is the complex metabolic heterogeneity of cancer cells. The efficacy of ES is dependent on mitochondrial function. Tumors that rely on oxidative phosphorylation are more sensitive to ES‐induced oxidative stress. Tumors that rely on glycolysis are less affected, but those that rely on oxidative phosphorylation are more vulnerable to ES‐induced oxidative stress. This complexity makes it difficult to achieve consistent efficacy across many cancer types or even among subgroups of a single cancer type, such as melanoma, without appropriate patient selection based on tumor metabolism. In a phase III melanoma trial, patients with normal baseline lactate dehydrogenase (LDH) levels had a significant increase in progression‐free survival. This metabolic variance means that patient selection is critical. ES did not significantly improve progression‐free survival (PFS) in unselected patient populations throughout phase III trials. For example, in the SYMMETRY study, while preclinical and early‐phase research revealed that ES could improve paclitaxel efficacy by inducing oxidative stress and mitochondrial death, these effects did not transfer into significant clinical benefits for all patients. The PFS was not significantly different between patients who received ES with paclitaxel and those who received only paclitaxel. This deficiency underscores the need to select appropriate patient populations based on biomarkers that predict therapy response [23, 27, 31].

#### 4.2.2 Safety and Survival Concerns

Safety concerns were among the most common causes of ES trial failure. The phase III SYMMETRY trial, which tested ES in combination with paclitaxel for metastatic melanoma, showed higher mortality in the combination therapy group than in the paclitaxel‐only group, prompting the trial's suspension. This raised serious concerns about ES's safety profile when combined with other chemotherapeutic medicines, particularly in individuals with high lactate dehydrogenase (LDH) levels, which indicate an aggressive illness. A phase II ovarian cancer trial demonstrated adequate tolerability but was only modestly effective. The study reported a 19.6% objective response rate [20, 23, 27].

#### 4.2.3 Biomarker Limitations

Predictive biomarkers are one of the key focus areas. Low LDH levels associated with better melanoma outcomes suggest a link to mitochondrial metabolism. This link was less obvious in acute myeloid leukemia, indicating the need for more advanced patient classification tools. In many clinical tests, ES showed high patient tolerance. However, one clinical investigation found that the ES and paclitaxel combined group had a greater death rate. The clinical trial was terminated. Patients with raised LDH levels died at a greater rate when treated with ES and paclitaxel in combination. It indicated that the efficacy of ES may be limited to specific patient subpopulations and that the studies lacked appropriate biomarkers for patients who might respond positively to the treatment. Future studies on combination therapy may highlight the significance of LDH levels in patients [23, 27].

#### 4.2.4 Pharmacokinetics Consideration

ES is practically insoluble in water and it has been developed as a sodium salt for its clinical use. ES is rapidly eliminated from plasma and has a short half‐life, so maintaining the therapeutic levels is challenging [20]. The solubility and formulation of drugs can also affect dose administration and therapeutic use. There is a possibility that new delivery technologies, such as nanoparticles, could overcome difficulties and increase efficiency.

#### 4.2.5 Combination Therapy Challenges

Combining ES with other medications that complement its activity may increase its effectiveness since it targets mitochondrial metabolism and ES mechanisms for ROS production. A number of studies have found that ES might be more potent when combined with glycolysis inhibitors, platinum compounds, or proteasome inhibitors. The failure of the initial research prompted a rethinking of combination techniques designed to utilize ES's unique mechanism [27, 42].

### 4.3 Suggestions for Future Studies

#### 4.3.1 Patient Selection Based on Biomarkers

According to the phase 3 melanoma trial, patients with high LDH levels had worse outcomes when treated with ES. Future studies should consider biomarkers to identify subpopulations that are more likely to respond to the treatment. As an example, patients with low LDH levels and tumors that depend heavily on mitochondria might respond better to ES treatment [27].

#### 4.3.2 Targeting Certain Metabolic Profiles

Copper ion transport and mitochondrial metabolism are key to the mechanism of ES‐induced cell death. Preclinical studies show that tumors with high OXPHOS activity are more likely to respond to ES‐induced cuproptosis. Future research should thus concentrate on cancers that rely on mitochondrial metabolism. Metabolomic profiling and other metabolic vulnerability detection in cancer cells can be used to personalize ES treatments to specific patient groups. Determined biomarker screening, such as LDH levels or metabolic profiles, should be included in future clinical trials to help identify patient subgroups who will benefit most from the treatment. This may result in more targeted treatment, which could improve overall outcomes [27, 31].

#### 4.3.3 Improved Formulations for Optimal Efficacy

ES's limited water solubility and rapid excretion make it difficult to maintain therapeutic concentration in patients. Preclinical studies using nanomedicine‐based formulations, such as micellar systems or lipid‐based nanoparticles, have shown promise for improving intracellular delivery and drug stability. Future studies should focus on developing nano‐formulations to improve pharmacokinetics and therapeutic outcomes further in clinical research [43, 44].

#### 4.3.4 Combination Therapies

Combining ES with other treatments, such as ferroptosis inducers (e.g., sorafenib) or MEK inhibitors (e.g., binimetinib), has shown promising results in preclinical studies. Future studies should take these combined strategies into consideration, especially for tumors where ES has shown limited efficacy [45].

Despite the fact that many ES clinical studies did not meet their primary objectives, their limitations provide valuable information. Research should focus on developing new formulations to improve drug delivery, combination therapies that induce cuproptosis, and improving patient selection through biomarker‐driven approaches. The consideration of these factors may contribute to improved clinical outcomes and enhance the therapeutic potential of ES for cancer treatment.

## 5 ES Formulation and Delivery in Various Cancers and Its Practical Implications and Challenges in Clinical Application

### 5.1 ES Sodium

ES sodium is a water‐soluble sodium salt. It has been used in several clinical trials. The clinical studies using ES sodium have been discussed before in this review. In most of the preclinical studies, ES was dissolved in DMSO and then further dosed to various cell lines. In a study reported by Lee et al., ES stock was dissolved in DMSO at 20 mM and further diluted with DMSO for the experimental study. ES monotherapy had no effect, but genipin modestly decreased 18F‐fluorodeoxyglucose (FDG) uptake while increasing cellular and mitochondrial ROS generation. The combination of ES and genipin showed significantly enhanced results. The increased ROS generation and decreased glucose uptake were due to the inhibition of UCP2 activity‐mediated oxidative stress protection. The combination of ES and genipin significantly decreased A549 cell mitochondrial membrane potential and could trigger apoptosis, indicating that UCP2 protects cancer cells against ROS‐induced apoptosis via the intrinsic apoptotic pathway. In vivo studies showed consistent results, suggesting that suppressing UCP2 activity with genipin could reduce glucose uptake, increase mitochondrial ROS generation, and improve cytotoxic effects in cancer cells [46].

ES has been reported by Buccarelli et al. that it could impair glioblastoma stem‐like cell (GSCs) survival and tumor growth by inducing mitochondrial ROS generation. In this study, researchers screened an anticancer drug library and evaluated the effect on cellular signaling pathways by a (phospho‐) proteomic analysis (reverse‐phase protein arrays [RPPA]) in order to find the most effective drug that could inhibit the survival pathways of both GSCs and GSC‐derived endothelial cells (GdECs). ES was dissolved in DMSO for the treatment in cells. The screening result showed that both the GSCs grown in stem cell medium and those cultivated under endothelial medium showed a high degree of sensitivity to ES. The researchers further proved that ES could inhibit both GSCs and GdECs and induced non‐apoptotic copper‐dependent cell death mainly due to the mitochondrial ROS induced by ES. This study also shows that the combination of ES with TMZ improves the anticancer effect of TMZ alone in vitro and in vivo, indicating that addressing oxidative stress could be a promising strategy for new therapeutics in glioblastoma [47].

### 5.2 ES–Cu Nanomedicine

**FIGURE 7:**
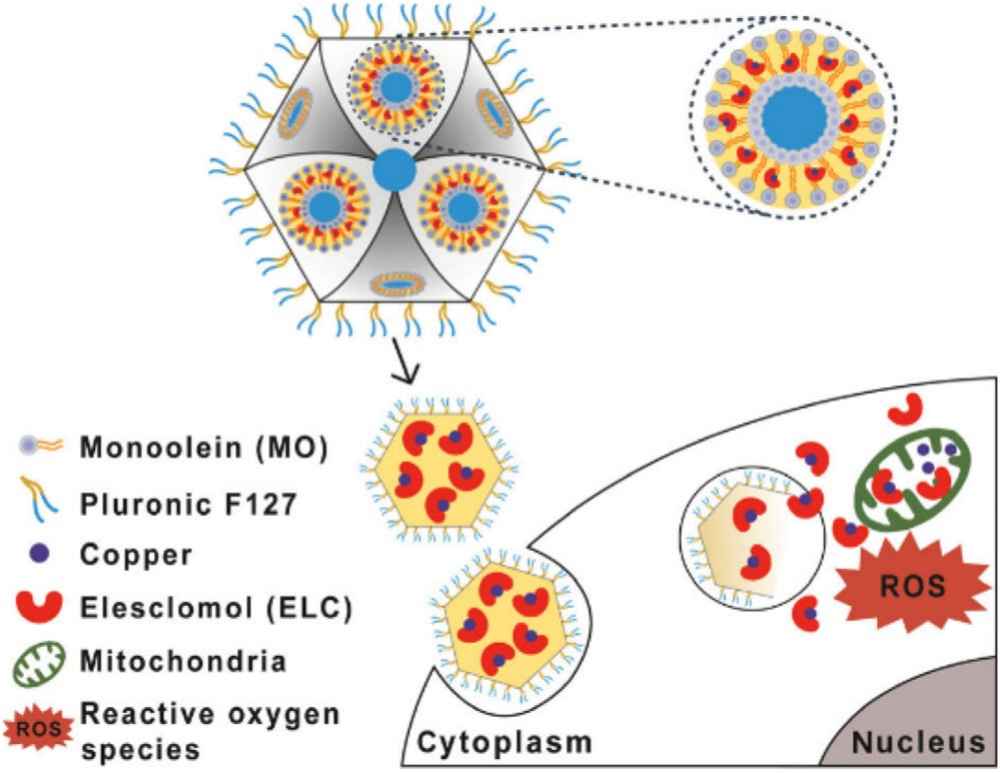
The illustration of ES–Cu loaded cubosomes. Reprinted with the permission of Ref. [44]. Copyright Tsinghua University Press and Springer‐Verlag GmbH Germany, part of Springer Nature 2018.

It has only been reported in a few research papers that ES–Cu complex can be delivered via nanoparticles. For example, Faria et al. were the first to report encapsulating ES or ES–Cu complex in a lipid‐based nanocarrier (Figure 7). The researchers used monoolein and Pluronic F127 to fabricate a cubosome nanoparticle, named ELC‐Cub, in order to load ES or ES–Cu complex. The results showed that the ELC‐Cub had suitable physiochemical properties that were promising for future systemic administration. The cytotoxicity of ELC‐Cub in A549 and A431 cancer cell lines was tested. The researchers also compared the EC50 values between ELC‐Cub and free ES–Cu complex in culture medium. ELC‐Cub showed a significantly higher anticancer efficacy than free ES–Cu complex in cells. This study screened a suitable cubosome formulation for ES–Cu complex encapsulation and could provide a potential delivery strategy for ES–Cu complex in nanoparticle systems [44].

**FIGURE 8:**
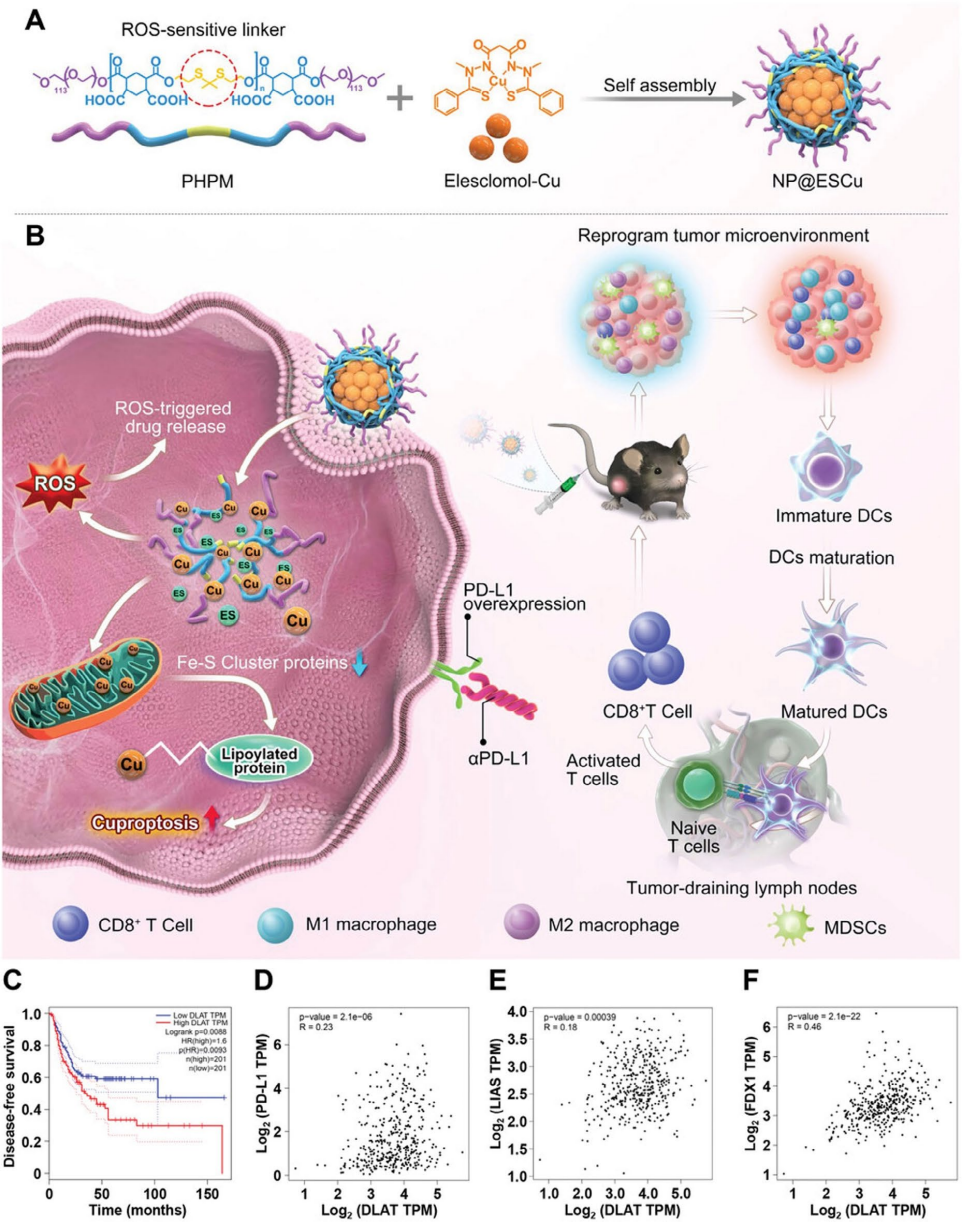
The illustration of NP@ESCu nanoparticles. Reprinted with permission of Ref. [48]. Copyright 2023 Wiley‐VCH GmbH.

Guo et al. developed a ROS‐sensitive nanoparticle to co‐deliver ES and copper (NP@ESCu). The NP@ESCu exhibited good stability and induced cuproptosis (2023), as shown in Figure 8. By performing RNA‐seq, the researchers reported that the NP@ESCu could downregulate the expression of the cuproptosis‐related markers of Fe‐S cluster genes ACO2 and POLD1 while activating the cytokine‐cytokine receptor interaction pathway in cancer cells. They also found that NP@ESCu could promote DC maturation, enhance CD8+ T cell infiltration, improve TME, and significantly increase PD‐L1 expression in cancer cells. By combining NP@ESCu with αPD‐L1, they reported there was a synergistic effect with enhanced efficacy in immune therapy. This study not only proposed a novel ROS‐sensitive nanoparticle to co‐deliver ES–Cu, but also provided a promising administration strategy for cancer therapy with enhanced immune efficacy [48].

**FIGURE 9:**
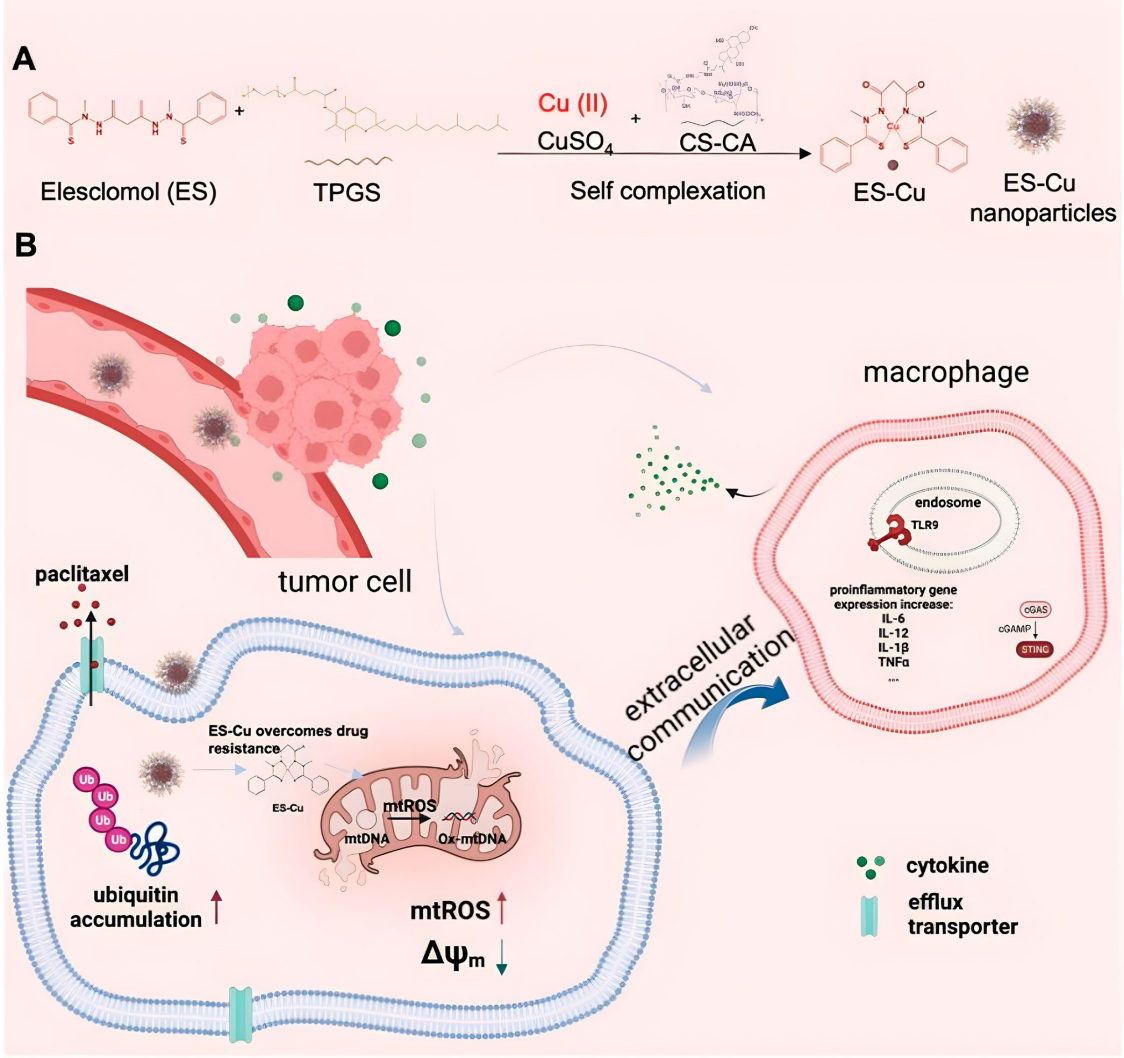
The illustration of ES‐Copper nanoparticles. Reprinted with the permission of Ref. [43]. Copyright 2024, American Chemical Society.

Recently, Wang et al. developed d‐α‐tocopherol polyethylene glycol 1000 succinate (TPGS) and chondroitin sulfate–cholic acid (CS–CA) based TPGS/CS–CA micellar nanoparticles to deliver ES–Cu complex into cancer cells, as shown in Figure 9. The ES–Cu complex was synthesized before nanoparticle preparation in the first two studies. In the first two studies of ES and copper nanoparticle delivery systems, a first step of synthesizing the ES–Cu complex was required before preparing the nanoparticles. In addition, complex steps were required for the nanoparticle preparation. Easy preparation methods are needed for ES and copper codelivery. In this study reported by Wang et al., the ES–Cu complex was not synthesized before the preparation. Instead, ES can self‐complex with copper to form an ES–Cu complex during the preparation step. The nanoparticles showed high encapsulation efficiency and stability. The ES–Cu in a nanoparticulate formulation facilitated the intracellular delivery of both actives (ES and Cu). The poor solubility of the ES–Cu complex makes it difficult to deliver to cancer cells. The nanoparticles effectively encapsulated ES–Cu and provided a targeted delivery system. This system showed a high efficacy with anticancer effects in various cancer cell lines such as DU145, PC3, A549, and the corresponding drug‐resistant cell lines (DU145TXR, PC3TXR, and A549TXR). This system was found to bypass the P‐glycoprotein (P‐gp) transporter with more beneficial results and with potential for overcoming drug resistance. Additionally, the nanoparticles induced M1 polarization in macrophages, which may potentially stimulate an innate immune response, indicating a dual therapeutic approach [43]. This ES–Cu nanoparticle delivery system was easy to prepare and showed promising results in cancer therapy.

**FIGURE 10:**
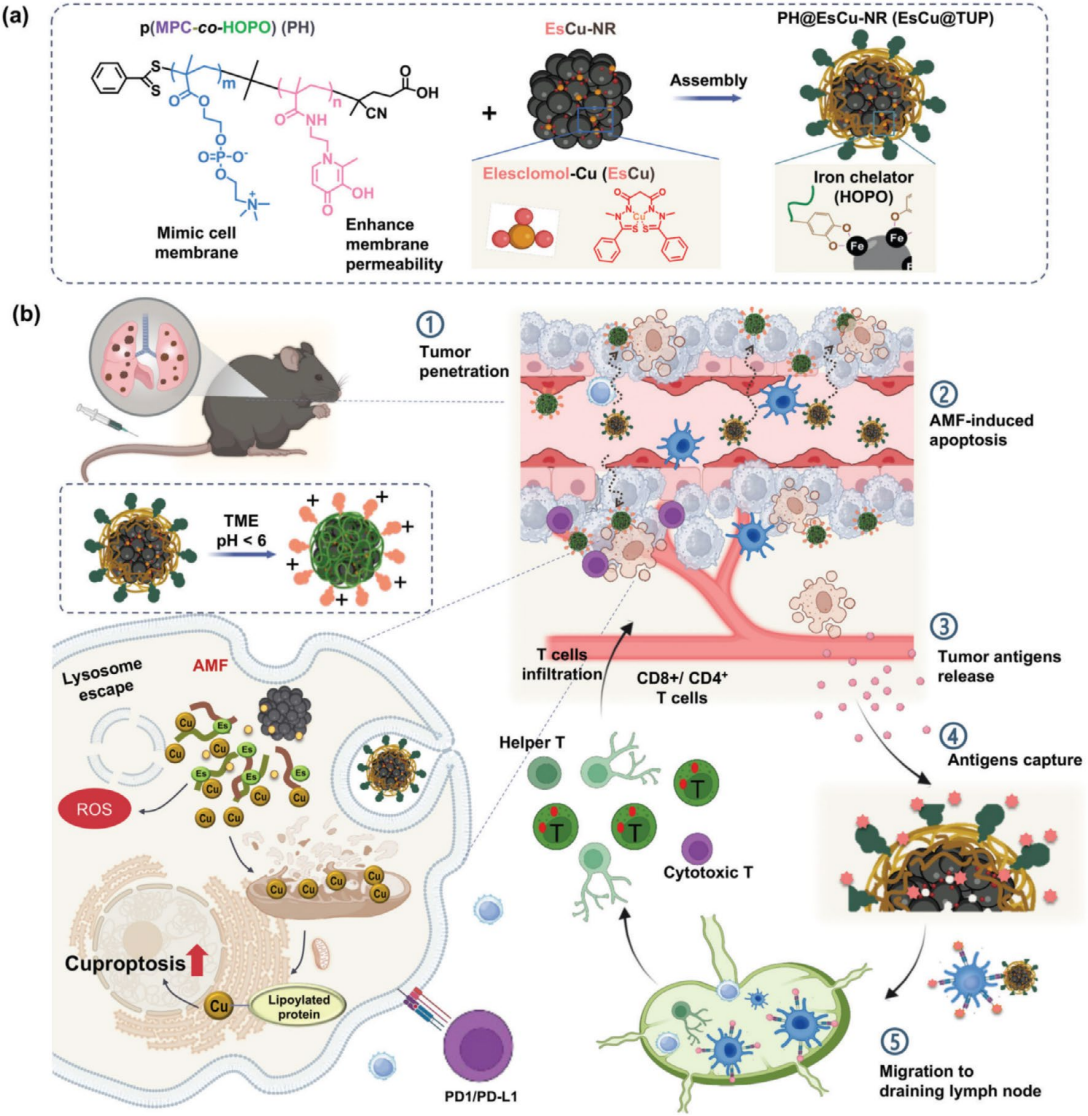
The schematic of EsCu@TUP2 formation and its mechanism of inducing cuproptosis and immune response. Reprinted with permission of Ref. [49]. Copyright 2024 Wiley‐VCH GmbH.

Huynh et al. developed ES–Cu (EsCu) nanoparticles encapsulated in tumor‐penetrating magnetic particles (TUP) for lung metastasis immunotherapy (2024), as shown in Figure 10. These nanoparticles (EsCu@TUP) were formulated with a cascade‐responsive cell membrane‐mimetic copolymer composed of zwitterionic 2‐methacryloyloxyethyl phosphorylcholine and 3‐hydroxypyridin‐4‐one (PH), along with magnetic nano raspberry (NR) cores. The nanoparticles exhibited excellent stability with particle sizes of 457 ± 24.5 nm for EsCu@TUP1 and 471.5 ± 28.67 nm for EsCu@TUP2, maintaining colloidal stability for up to 7 days. The PH coating enhanced EsCu nanoparticles biocompatibility, prolonged circulation time, and facilitated targeted delivery to tumors via charge conversion and hyperthermia effects. The EsCu nanoparticles induced cuproptosis while simultaneously promoting the release of tumor‐associated antigens (TAAs) to stimulate immune responses. The TUP accumulates in tumors through charge conversion of PH and hyperthermia effects, where ES and Cu are released in response to intracellular environments and heat. This dual‐action therapy reprogrammed the tumor microenvironment by enhancing T‐cell infiltration, activating dendritic cells, and prolonging immune stimulation. The study demonstrated that these nanoparticles effectively targeted lung metastases, improved immune activation, and prevented metastatic tumor progression, offering a novel strategy for cancer immunotherapy [49].

**FIGURE 11:**
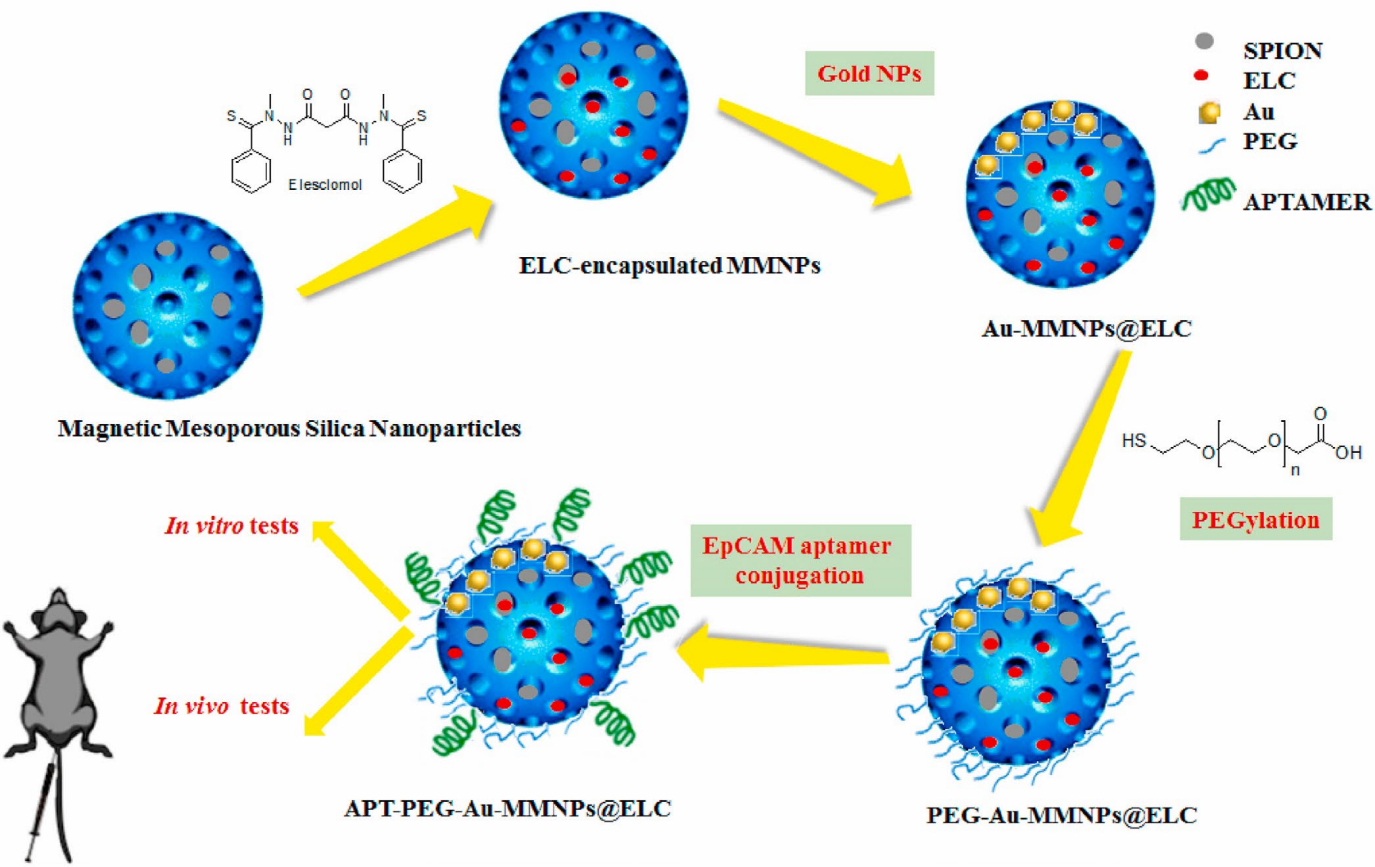
The schematic of APT‐PEG‐Au‐MMNPs@ELC preparation. Reprinted with permission of Ref. [50]. Copyright 2023 Elsevier B.V.

Metal–organic framework (MOF) has also been used to formulate ES–Cu nanoparticles. Lu et al. recently reported incorporating ES into a targeted theragnostic nanoparticle system (APT‐PEG‐Au‐MMNPs@ELC) designed for prostate cancer treatment (Figure 11). The formulation consisted of magnetic mesoporous silica nanoparticles (MMNPs) loaded with ES, with gold nanoparticles acting as gatekeepers, and surface modification using polyethylene glycol (PEG) and EpCAM aptamer for targeted delivery. The final nanoparticles had an average diameter of 81.13 ± 7.41 nm and demonstrated pH‐dependent sustained drug release ability over 72 h. The targeted nanoparticles showed enhanced cytotoxicity against prostate cancer (PC‐3) cells compared to normal cells, indicating improved selectivity. The nanoparticle delivery system effectively induced apoptosis in PC‐3 cells, and in in vivo studies using a PC‐3 xenograft tumor model, demonstrated significant tumor growth inhibition while reducing side effects of ELC. The efficacy could be attributed to the APT‐PEG‐Au‐MMNPs@ELC targeted delivery mechanism and the incorporation of both therapeutic and diagnostic capabilities in a single delivery system [50].

**FIGURE 12:**
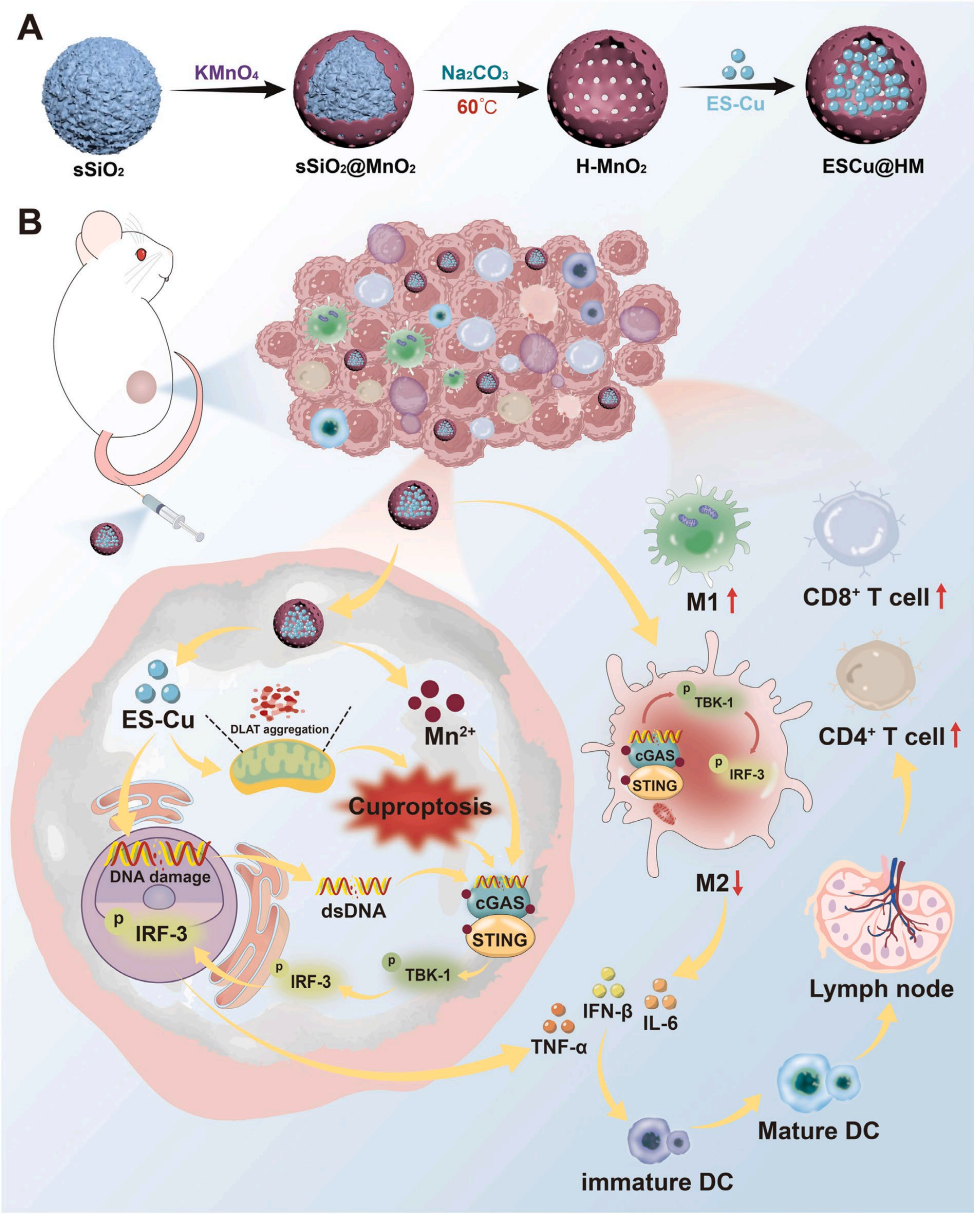
The schematic of the preparation and mechanism of ESCu@HM. Reprinted with permission of Ref. [51]. Copyright 2024 The Author(s). Published by Elsevier Ltd.

Li et al. developed a copper‐loaded nanoreactor, ESCu@HM, which was designed to enhance colorectal cancer (CRC) treatment by inducing cuproptosis and activating antitumor immunity (Figure 12). The ESCu@HM nanoreactor was demonstrated to be a novel therapeutic approach combining cuproptosis and immunotherapy for colorectal cancer treatment. The ESCu@HM nanoreactor consisted of hollow manganese dioxide (H‐MnO_2_) nanoparticles loaded with ES and copper ions (Cu^2+^) and showed excellent stability with a uniform size of approximately 120 nm. The acidic tumor microenvironment could trigger ESCu@HM to release Cu^2+^ and ES, and the released Cu^2+^ and ES could further trigger mitochondrial dysfunction and proteotoxic stress. The ESCu@HM nanoreactor damaged mitochondrial DNA, activated the cGAS‐STING immune pathway, and reprogrammed the tumor microenvironment by converting immunosuppressive macrophages (M2) into immunostimulatory macrophages (M1). In in vitro studies, ESCu@HM showed superior cellular uptake, significant copper accumulation, and enhanced cytotoxicity. In in vivo studies, it effectively inhibited tumor growth in mouse models, improved immune responses, and showed minimal toxicity to normal tissues. ESCu@HM offered a promising dual therapeutic strategy for CRC by combining targeted cuproptosis induction with immunotherapy [51].

**FIGURE 13:**
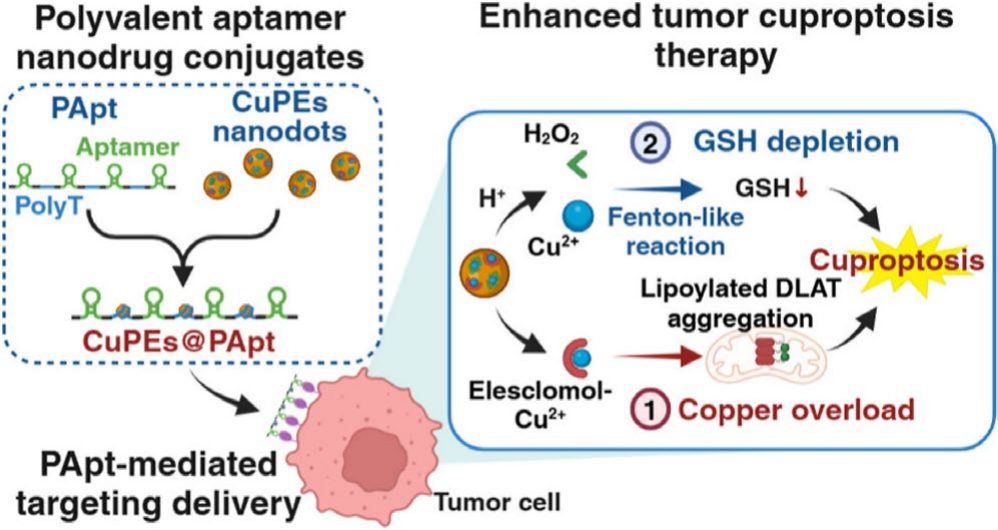
The schematic of CuPEs@PApt inducing Fenton‐like reaction, cuproptosis and immune response for cancer therapy. Reprinted with permission of Ref. [52]. Copyright 2024 American Chemical Society.

Wang et al. recently developed polyvalent aptamer‐nanodrug conjugates (CuPEs@PApt) with a nucleosome‐like structure for enhanced tumor cuproptosis therapy (Figure 13). CuPEs@PApt consisted of long‐stranded DNA containing epithelial cell adhesion molecule (EpCAM) aptamers for tumor targeting and PolyT sequences for copper ion (Cu^2+^) chelation, which facilitates the loading and delivery of copper peroxide‐ES nanodots (CuPEs). Through internalization by tumor cells, ES transported both exogenous and endogenous Cu^2+^ into mitochondria, where they were reduced to (Cu^+^), inducing cuproptosis by binding to lipoylated DLATs in the TCA cycle, leading to their oligomerization and triggering proteotoxic stress. Additionally, lysosomal degradation of CuPEs released Cu^2+^ and hydrogen peroxide (H_2_O_2_), catalyzing a Fenton‐like reaction that generated hydroxyl radicals (•OH) to deplete intracellular glutathione (GSH). This depletion enhanced mitochondrial copper overload and prevented GSH from chelating Cu^+^, thereby amplifying cuproptosis. The therapy also induced immunogenic cell death (ICD), activating antitumor immune responses. CuPEs@PApt significantly increased intracellular Cu^2+^ concentrations, depleted GSH levels, and reduced free lipoylated DLAT, LIAS, and FDX1 proteins, effectively inducing cuproptosis in vitro and in vivo. This “open‐source cost‐saving” strategy synergistically disrupted tumor copper homeostasis, offering a promising approach for precise and efficient cancer treatment with minimal drug resistance [52].

In summary, ES has been incorporated into various nanoparticle systems such as lipid‐based cubosomes, micelles, metal–organic frameworks (MOFs), and magnetic particles. Most formulations aim to enhance anticancer efficacy by inducing cuproptosis or improving immune responses. Some formulations also exhibit dual therapeutic effects by combining targeted delivery with immunotherapy. Common disadvantages include complex preparation methods, limited in vivo validation for certain formulations, and potential off‐target effects or toxicity. Table 5 provides a comprehensive overview of ES formulations and nanomedicines in preclinical research.

**TABLE 5:** A comprehensive summary of the current ES nano‐formulations and their applications.

Nano‐formulation	Composition	Characterization techniques	Cancer models	Advantages	Disadvantages
ELC‐Cub [44]	Lipid‐based cubosome nanoparticle with monoolein and Pluronic F127 encapsulating ES or ES–Cu complex	Particle size, zeta potential, encapsulation efficiency, cytotoxicity assays (EC50), in vitro cancer cell tests	A549, A431 cell lines (in vitro)	High anticancer efficacy compared to free ES–Cu; suitable physicochemical properties for systemic delivery	Limited to in vitro studies; no immune‐related effects explored
NP@ESCu [48]	ROS‐sensitive nanoparticles co‐delivering ES and copper	RNA‐seq, stability tests, immune pathway activation studies	In vitro cancer cells; combination with αPD‐L1 in vivo models	Synergistic immune therapy with αPD‐L1; improved tumor microenvironment (TME); enhanced CD8+ T‐cell infiltration	Complex preparation
TPGS/CS‐CA based ES–Cu nanoparticles [43]	TPGS and chondroitin sulfate‐cholic acid micellar nanoparticles delivering ES–Cu complex	Encapsulation efficiency, stability tests, cytotoxicity assays	DU145, PC3, A549 and drug‐resistant cell lines (in vitro)	Easy preparation method; bypasses P‐glycoprotein transporter; dual therapeutic effect (anticancer + immune stimulation)	No in vivo validation
EsCu@TUP [49]	Tumor‐penetrating magnetic particles with zwitterionic copolymer coating	Particle size, colloidal stability, immune activation assays	Lung metastasis model (in vivo)	Enhanced biocompatibility; prolonged circulation; dual‐action therapy targeting metastases	Larger particle size (~457 nm)
MOF nanoparticules [50]	Magnetic mesoporous silica nanoparticles with gold nanoparticle gatekeepers and PEG/EpCAM aptamer for targeted delivery	Particle size, pH‐dependent drug release studies, in vivo tumor growth inhibition	PC‐3 xenograft tumor model	Targeted delivery; theragnostic capabilities; reduced side effects	Complex synthesis process
ESCu@HM nanoreactor [51]	Hollow manganese dioxide nanoparticles loaded with ES and Cu^2+^	Cellular uptake studies, mitochondrial dysfunction assays, cGAS‐STING pathway activation	Colorectal cancer models (in vitro/in vivo)	Combines cuproptosis induction and immunotherapy; reprograms tumor microenvironment	Potential toxicity concerns due to MnO_2_ byproducts
CuPEs@PApt [52]	Polyvalent aptamer‐nanodrug conjugates with nucleosome‐like structure	Copper ion chelation studies, Fenton‐like reaction analysis, mitochondrial copper overload studies	In vitro and in vivo cancer models	Precise tumor targeting via aptamers; synergistic disruption of tumor copper homeostasis	Complex preparation steps

### 5.3 ES In Combination With CuO Nanoparticles

**FIGURE 14:**
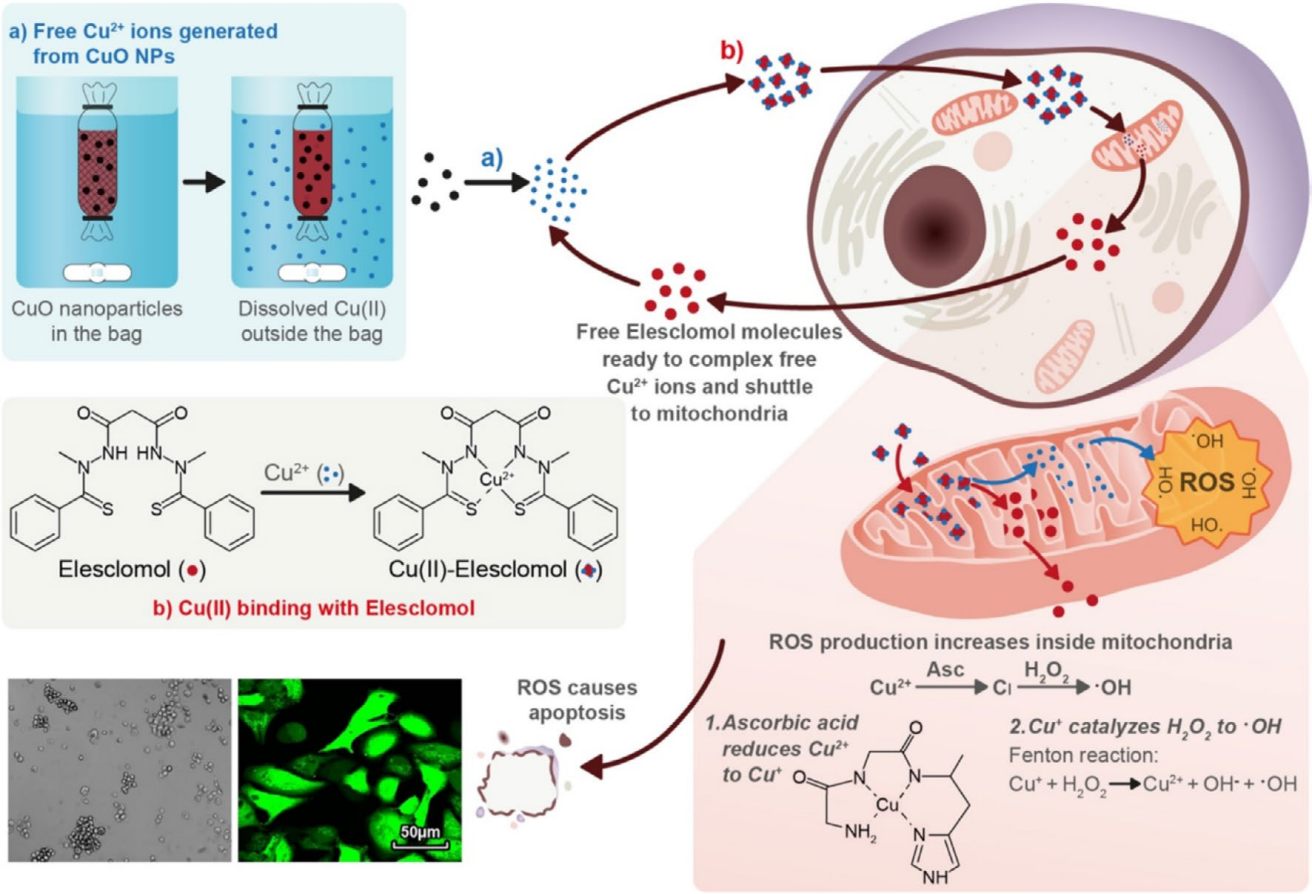
The schematic of Cu(II) release from CuO nanoparticles and ES delivers Cu(II) into mitochondria. Reprinted with the permission of Ref. [53]. Copyright 2023 Wiley‐VCH GmbH.

**FIGURE 15:**
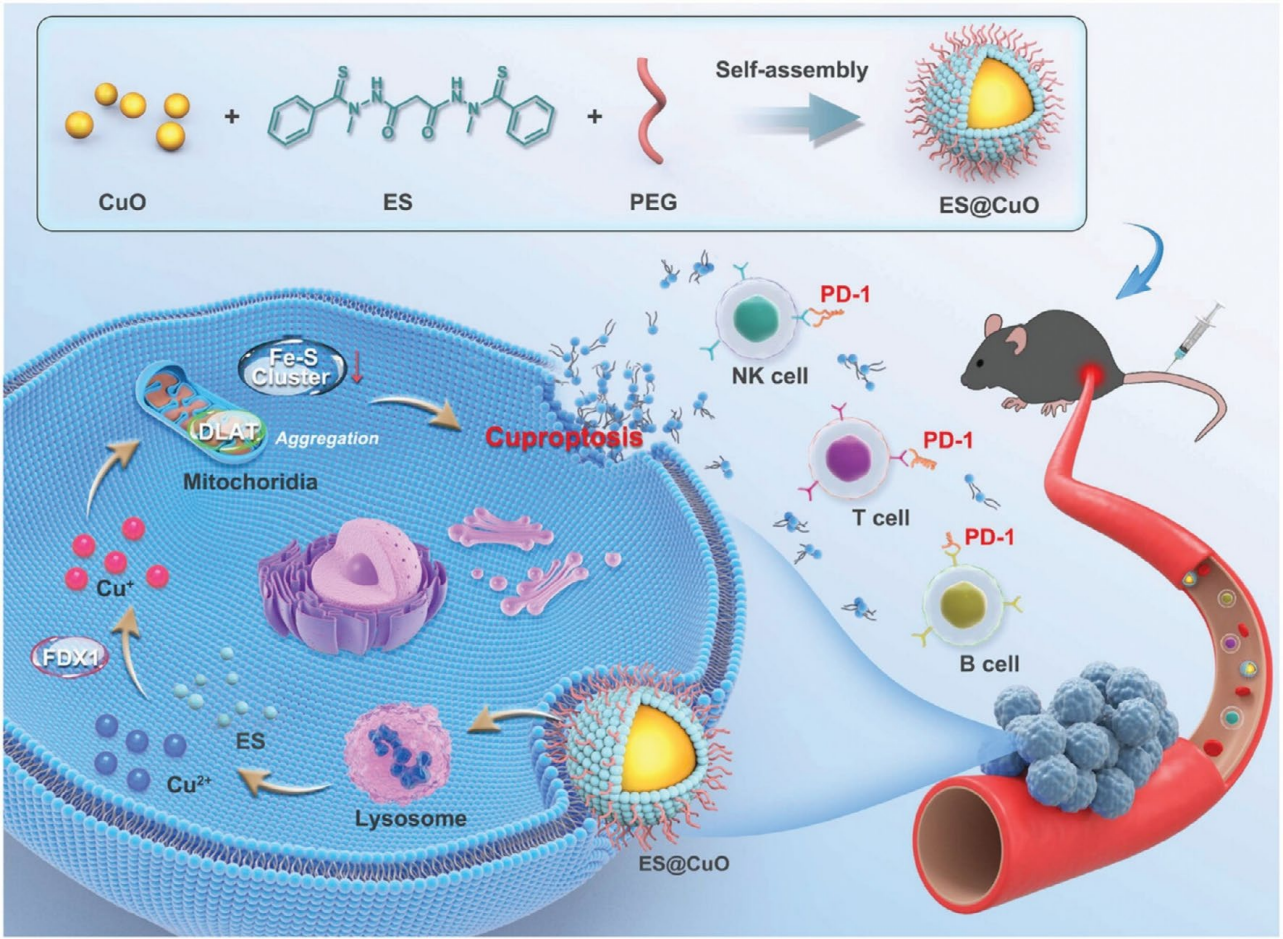
The schematic of ES@CuO preparation and its combination with PD‐1 to enhance anticancer efficacy. Reprinted from Ref. [54]. Copyright 2024 The Authors. Advanced Science published by Wiley‐VCH GmbH.

Few studies have shown ES–CuO nanoparticle delivery systems; for example, Chakraborty et al. developed a CuO nanoparticle delivery system in combination with ES for anticancer therapy [53]. Cu(II) can be released from CuO nanoparticles and then further bind to ES in cell culture media, as shown in Figure 14. When co‐delivered with ES, CuO nanoparticles significantly decreased the cell viability of A549 cells compared to the treatment of CuO nanoparticles alone. The intracellular accumulation of copper inside A549 cells increased by up to four times when the cells were treated with 1000 ng/mL CuO nanoparticles with 50 ng/mL ES. The study showed that CuO nanoparticles could be used as a Cu(II) reservoir with slow and sustained release of Cu (II) ions to bind with ES. The formation of Cu–ES then promoted intracellular oxidative stress. This combination administration strategy could be used as a promising approach for ES‐based anticancer therapy. This study also demonstrated that only the released Cu(II) could be shuttled inside the cells through ES. The codelivery system of CuO nanoparticles and ES showed the potential to induce cell death by triggering oxidative stress in anticancer therapy [53]. In another study, Lu et al. recently developed a pH‐responsive and cuproptosis‐inducing delivery system containing CuO and ES in one nanoparticle (ES@CuO), as shown in Figure 15 [54]. When entering cells, ES@CuO was degraded to release Cu(II) and ES, which then triggered cuproptosis in murine B16 melanoma cells. ES@CuO further triggered an immune response by inducing immune cell death. When combined with programmed cell death‐1 (PD‐1) immunotherapy, the efficacy increased compared to the treatment of ES@CuO alone. This ES@CuO nanoparticle delivery system provides novel strategies for cuproptosis‐mediated anticancer therapy, which then enhances the efficacy of immune checkpoint inhibitor therapy.

### 5.4 ES Practical Implications and Challenges in Clinical Application

#### 5.4.1 Practical Implications

In addition to enhancing ES's stability, targeting, and therapeutic efficacy, nanoparticle‐based delivery can be used in combination therapy to improve cancer treatment while minimizing off‐target effects and toxicity. This includes:

- Enhanced delivery and targeting: Nanoparticles can improve ES bioavailability by protecting it from early degradation, improving its solubility, and allowing for regulated release, thereby expanding its practical applications. By allowing targeted distribution to tumor sites, it also helps to reduce off‐target effects and toxicity [31, 55].
- Combination therapies: Combination therapy can improve anticancer activity by co‐delivering ES with other therapeutic drugs such as chemotherapeutics or glycolysis inhibitors [31].

Precision medicine: Nanoparticle development leads to patient‐specific applications by including biomarkers or targeting moieties that react to specific tumor sites [55].

#### 5.4.2 Difficulties in Clinical Translation

For clinical translation of the findings, it is important to consider the following factors.

- Biological barriers: Nanoparticles must overcome biological barriers such as immune clearance, varied tumor microenvironment, and limited tumor penetration. These factors may reduce the effectiveness of nanoparticle‐based delivery systems [55, 56].
- Safety concerns: Nanoparticles may accumulate in non‐target tissues as a result of biodistribution, potentially causing side effects. Furthermore, the safety of new excipients used in nanoparticle formulations must be thoroughly evaluated [56, 57].
- Manufacturing and scalability: Nanoparticles are challenging to manufacture and scale due to their complicated synthesis and characterization. A major problem is establishing batch‐to‐batch homogeneity while adhering to Good Manufacturing Practice (GMP) guidelines [57, 58].
- Regulatory difficulties: Because nanomedicines are still being developed, approval processes remain uncertain. Researchers should focus on unique quality control parameters such as particle size, surface charge, and drug release properties [57, 58].
- Translating preclinical models: Many nanomedicine candidates demonstrate promising results in animal studies but fail in clinical trials due to differences in nanoparticle behavior between animal models and humans. Better predictive preclinical models will assist in narrowing this gap [58].

#### 5.4.3 Strategies to Address Challenges

Using technology such as microfluidics or coaxial turbulent jet mixers will improve the repeatability and scalability of nanoparticle production. Extensive preclinical research on biodistribution and organ‐specific toxicity assists in addressing safety concerns through early toxicology studies [56, 57, 58]. Biomarkers can be used to select patient populations and choose those most likely to benefit from ES‐based treatments, improving the clinical outcomes [31, 55, 58].

In summary, while nanomedicine‐based approaches to deliver ES have immense promise to improve cancer therapy results, their successful clinical use is contingent on overcoming scientific, manufacturing, safety, and regulatory constraints. The translation of these breakthroughs into practical applications will be dependent on overcoming these obstacles through inventive technologies.

## 6 Conclusion

ES, a copper ionophore, has shown great potential in cancer treatment. The use of ES in targeting proteasome inhibitor‐resistant cancer cells, causing oxidative stress, and modifying mitochondrial metabolism has been discussed. Current application challenges include safety concerns in combination therapy and modest progression‐free survival benefits in ES. ES nano‐formulations have shown potential in increasing solubility, stability, and intracellular distribution, and improving therapeutic efficacy. Combining ES with ferroptosis inducers or immune checkpoint inhibitors provides synergistic strategies for overcoming drug resistance and increasing efficacy.

Future research should focus on optimizing ES formulations, developing combination strategies, and conducting well‐designed clinical trials with appropriate patient selection.

## Author Contributions

Conceptualization: Feng Li, R. Jayachandra Babu, Amit K. Tiwari. Funding Acquisition: Feng Li, R. Jayachandra Babu. Resources: Feng Li, R. Jayachandra Babu. Supervision: Feng Li, R. Jayachandra Babu. Writing – Original Draft Preparation: Qi Wang, R. Jayachandra Babu. Writing – Review and Editing: Qi Wang, Feng Li, Amit K. Tiwari, and R. Jayachandra Babu.

## Disclosure

The views and opinions expressed in this manuscript are those of the authors only and do not necessarily represent the views, official policy, or position of the U.S. Department of Health and Human Services or any of its affiliated institutions or agencies. A.K.T. is supported by Arkansas Bioscience Institute funds (ABI‐GR020025) from the University of Arkansas for Medical Sciences.

## Conflicts of Interest

The authors declare no conflicts of interest.

## Data Availability

The data that support the findings will be available in https://pubmed.ncbi.nlm.nih.gov/, https://scholar.google.com/, clinicaltrials.gov following an embargo from the date of publication to allow for commercialization of research findings.
